# Estimation of Blockage Position, Geometry, and Solidity in Molten Salt Pipelines

**DOI:** 10.3390/s20123490

**Published:** 2020-06-20

**Authors:** Alberto M. Pernía, Héctor Andrés Mayor, Miguel J. Prieto, Pedro J. Villegas, Juan Á. Martínez, Juan A. Martín-Ramos

**Affiliations:** Department of Electrical Engineering, University of Oviedo, 33204 Gijón, Spain; hector.andres@eu4m.eu (H.A.M.); mike@uniovi.es (M.J.P.); pedroj@uniovi.es (P.J.V.); jamartinez@uniovi.es (J.Á.M.); jamartin@uniovi.es (J.A.M.-R.)

**Keywords:** guided waves (GW), magnetostriction, magnetostrictive sensors (MsS), electromagnetic acoustic transducers (EMATs), non-destructive testing (NDT), molten salts, heat transfer fluid (HTF), thermosolar

## Abstract

In solar thermal plants, the use of molten salt as a heat transfer fluid is an advantageous alternative, although it has some disadvantages such as the formation of salt plugs in the pipes due to possible stratification of the salt or its solidification. The aim of this study was to implement an electromagnetic acoustic transducer (EMAT) not only capable of identifying the position of the plug, but also of determining whether the plug blocks the entire conductive surface or, on the contrary, is partial, allowing the fluid to pass through a smaller section. The proposed transducer is intended to be minimally invasive, allowing it to be used in the same way as a temperature probe. To do so, it creates torsional waves in the pipe, which are then used for a combination of measurements: pulse-echo and attenuation of the acoustic waves. Two materials with different densities (silicone and cement) were used in the tests carried out, which made it possible to check that for a given size of blockage, it is possible to identify the type of material from which it is formed.

## 1. Introduction

The European Union (E.U.) is committed to fighting against anthropogenic climate change by reducing greenhouse gas emissions by at least 40%, when compared to the 1990 levels, by 2030. One of the essential instruments to meet this demanding objective is the use of renewable energy. In this sense, the E.U. has agreed on a new binding target, raising the renewable share to at least 32% of the total energy consumption. Therefore, further development and market penetration of clean energy sources such as wind, photovoltaic, solar thermal, etc., are expected in the coming years.

The sun is a virtually endless source of energy and solar thermal power plants [1,2] have remarkable potential in the production and storage of renewable energy [3,4,5]. Its operation involves the use of parabolic trough solar collectors or Fresnel-type reflectors to heat a fluid to temperatures ranging usually from 200 °C to 400 °C [6]. Through one or more heat exchangers, this fluid eventually produces steam to generate electricity in a turbine. A remarkable feature of these plants is the ability to store energy thanks to the presence of storage tanks of high temperature fluids. In this way, even in the absence of solar radiation, it is possible to continue with energy production.

Although there are several alternatives for the heat storing fluids, molten salts in the form of a KNO_3_-NaNO_3_ eutectic solution with a proportion of 54% and 46% by weight, respectively, is one of the most commonly used mixtures in medium- and high-temperature plants. As notable features of this type of molten salt, their low cost and the fact that they are neither flammable nor toxic must be mentioned. It is also important to note that their melting point is around 222 °C; lower temperatures cause the salt to solidify.

Despite the back-up electric heating system incorporated around the pipes through which the molten salt flows, gradual stratification of the salt at certain points or possible partial freezing may occur. This progressive process can eventually cause the formation of clogs that block the pipe and paralyze the operation of the solar plant. Therefore, if partial blockages are located and identified in time, plant shutdown can be avoided, or at least shortened. In fact, the work of overheating and melting the blockages is only possible if the positions of their ends are well known. This is because the salt expands in the change from solid to liquid. Therefore, melting a blockage from the middle produces a pressure increment inside the clog, which could derive in pipe bursting. Melting salt pouring out of a pipe constitutes a considerable risk to personnel.

There is an additional difficulty in determining the position where the clog is formed: the high temperature of the pipe. Hence, the sensor proposed in this work was developed with materials that can withstand these high temperatures.

A well-known technique for detecting plugs in pipes is that based on generating pressure waves inside the pipeline [7,8,9]. This pressure wave travels through the fluid, causing bounces in the presence of blockages or liquid leaks. The results obtained with this technique, typically used in pressurized water pipelines, are remarkable. In thermal solar plants, however, where the fluid can reach temperatures higher than 300 °C, the use of this technology has not been tested. With such high temperatures, a technique that can detect blockages from outside the pipe is recommended.

Acoustic wave generation is a non-intrusive and also quite common technique that has allowed for the detection of defects in pipes in numerous applications (petrochemical industries, for instance) during the last 20 years [10,11,12,13]. This technique is based on waves that travel along the pipe and bounce off those defects, thus providing information that can be analyzed.

Electromechanical devices used to generate these guided acoustic waves are typically based on piezoelectric materials [13], which are again limited to low-temperature applications of up to 150 °C.

Electromagnetic acoustic transducers, EMATs, [14,15] are another interesting alternative. These transducers work by causing the deformation of a metallic material, which can be the pipe itself or an additional element attached to it. This deformation can occur through two mechanisms: magnetostriction, based on the interaction of two magnetic fields, and Lorentz force, which uses the force produced by the interaction of some eddy currents and a magnetic field. Regardless of the mechanism chosen, the generated deformation is transmitted as an ultrasonic acoustic wave along the surface of the pipe to be examined. The rebounds generated from existing defects in the pipe (corrosion, cracks, etc.) are used to locate their position. Both alternatives have the advantage of allowing the use of materials that can withstand, a priori, temperatures of up to 700 °C.

Among the acoustic waves used to analyze the condition of the pipeline, guided waves have been widely used in the past years [11,12]. This technique allows for the inspection of pipelines over many tens of meters up to 120 m [16,17], and has therefore been proposed as capable of detecting blockages in the pipeline [18,19]. However, the guided-wave technique is not without its difficulties. One of these difficulties is caused by multimode propagation. A classification of guided waves was proposed by Meitzler in 1961 [20] that distinguished three main categories: asymmetric torsional axis T(0,m), asymmetric longitudinal axis L(0,m), and non-asymmetric flexural waves F(n,m). In each case, different modes can be distinguished (m = 1,2,3,4, ...).

The presence of several propagation modes simultaneously at a particular frequency complicates the analysis of the received signals. This is a typical problem with the guided-wave technique. However, the dispersive effects above-mentioned can be minimized by proper transducer design, signal processing, and inspection experience.

In this sense, wavelet-based alternatives [21] have been proposed for the correct filtering of the received signals. Rose [22] also proposed a multichannel transducer with a time delay system capable of incorporating delays in the generated signals from 0 to 30 µs; this study showed that this method succeeded in attenuating the unwanted modes with an improved sensitivity of the system.

All of these techniques try to maximize the signal/noise ratio, but the problem of unwanted modes is still inherent in this technology. This article is not devoted to the study and selection of the most appropriate type of guided wave, but an attempt has been made to minimize the problem by using an EMAT that tries to generate torsional mode waves T(0,1). This type of guided wave is particularly interesting in this application because of its non-dispersive characteristics with constant speed at any frequency, and because it is the only torsional mode at low frequencies.

In this scenario, the objective pursued in this work was not only to detect the obstruction, but also to determine if it covered the total section of the pipe. There is interest in industry to identify partial blockages in pipelines. In this situation, where the clog is not completely in contact with the entire inner wall of the pipe, torsional guided waves are an interesting alternative as an analysis mechanism, since they travel along the entire wall of the pipe during its advance [23,24,25].

For these purposes, a magnetostriction-based EMAT has been developed that uses permendur as the magnetic material [26,27]. By applying a variable magnetic field to the permendur strips, magnetostriction of the material occurs. If the strips come into contact with the pipe, the vibration is transferred and results in a guided wave along the pipe. The use of permendur strips enhances magnetostriction and allows for optimizing the design of the winding in charge of its magnetic polarization; since this material reduces the field strength needed to cause the deformation, a lower current is required from the winding responsible for generating the magnetic field. Additionally, the contact between the permendur strip and the pipe does not require high pressure and metal flanges are typically used for this purpose. In [19], the construction details of the magnetic head used for the magnetic polarization of the permendur strips can be found. This magnetic head, which produces torsional guided waves, will be the one used in the tests shown in Section 3.3.

Once the section of the pipeline affected by the clog is identified, there is still industrial interest in gathering more information about its geometry, edges, and consistency to properly start the melting process. In fact, the clog may not contact the entire inner wall of the pipe. By carrying out a second test close to the position of the clog, this paper demonstrates that it is possible to use the same technique for such a detailed study. In this case, measurements from a distance of a couple of meters (or even right over the clog) are possible and reasonable, just like those carried out in the experimental section of this work.

The rest of this paper is organized as follows. Section 2 presents the construction aspects of the proposed EMAT as well as details about its performance to obtain the position of the clog in a pipeline. Section 3 uses the same EMAT concept to focus on a closer study of the blockage and shows that more information about clog shape and consistency can be obtained. Finally, Section 4 analyzes and summarizes the results of the work to extract some conclusions.

## 2. Magnetostrictive Sensor for Blockage Detection

Some materials present the property of deforming under the effect of a magnetic field (Table 1). Magnetostrictive sensors are based on these materials. This is the case of permendur (cobalt–iron alloy 49Fe-49Co-2V), where the deformation reaches a value of ΔL/L = 70 µm/m under a field of the order of 600 Oe. The material can also be magnetically polarized to obtain a higher linear response.

**Figure 1:**
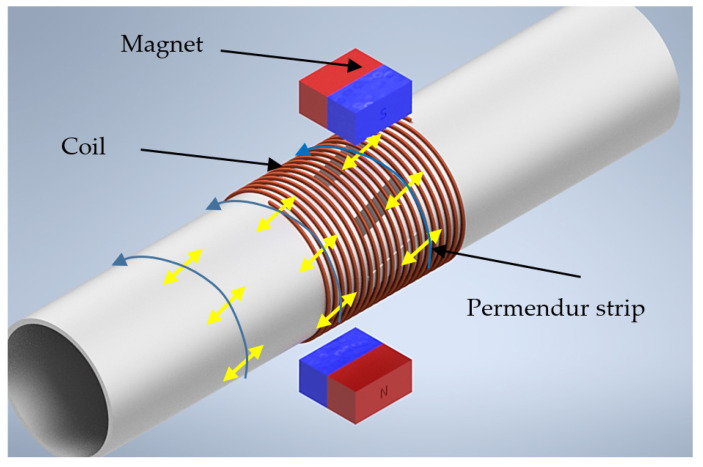
Magnetostrictive transducer (EMAT).

A sensor was assembled using this property. To do so, some permendur strips were firmly attached to a pipe and exposed to a variable magnetic field, which causes a deformation [28]. The deformation is transmitted to the surface of the pipe, causing an ultrasonic wave to be generated and propagated along the pipe. When the wave finds a defect, reflected echoes are generated, allowing its subsequent detection. Figure 1 shows the arrangement of the sensor on the pipe. The permendur strips were placed at a 45-degree angle, so that the deformation generated would cause a torsional wave to travel along the entire surface of the pipe [19,20].

**Figure 2:**
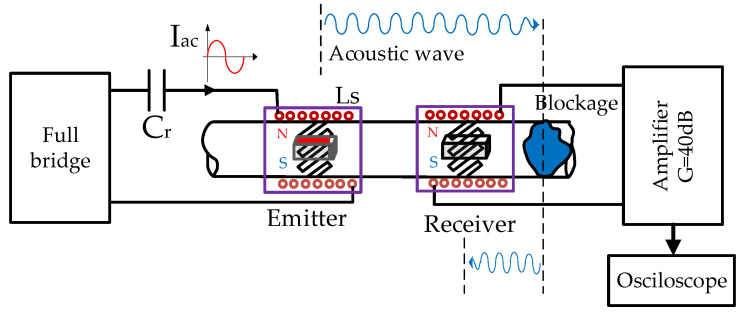
Experimental setup.

Figure 2 outlines the configuration of the experiment carried out. The structure of the emitter and the receiver was the same as described above. Both devices consisted of several parts. An external winding formed a solenoid around the pipe. This winding generated an alternating magnetic field that crossed the permendur strips placed at 45° and caused them to vibrate, generating torsional waves. In addition, two permanent magnets were placed on both sides of the pipe to create a continuous field that polarizes the permendur strips without having them reach saturation. This arrangement allows for greater variation in elongation and improved linearity between the magnetic field and the elongation. Using both sensors, and knowing the transmission speed of the generated wave, it is possible to detect the position of the blockage. In the case of aluminum pipes, this speed is of the order of 3120 m/s for torsional waves.

The emitter is activated with a full bridge inverter. This inverter drives a resonant circuit formed by capacitor Cr and the emitter winding inductance [29,30]. As a consequence of the resonance, the shape of the current through the solenoid is quasi-sinusoidal, generating the aforementioned alternating magnetic field with origin of the vibration in the permendur strips. Although the distance to the blockage considered in the tests may seem short, the EMAT technique can also be used for longer pipes, since guided waves have already proven their usefulness in travel spans of several tens of meters [17,18,27].

**Figure 3:**
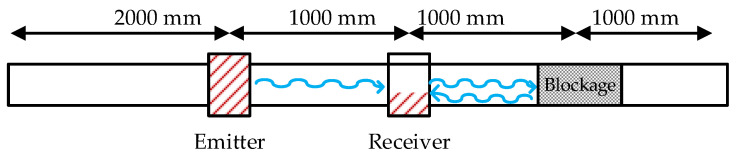
Distances between the emitter, receiver, and blockage.

**Figure 4:**
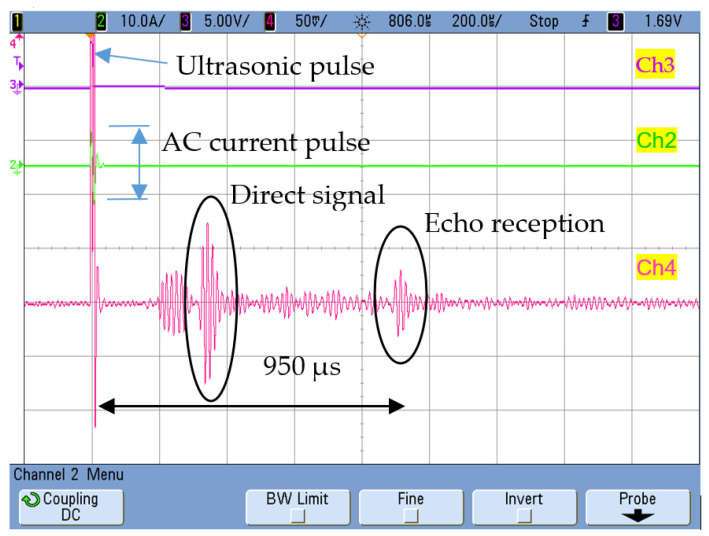
Ch1 [2 V/div]: Signal from the power stage. Ch2 [5 A/div]: resonant current. Ch4: [50 mV/div]: Bounce detection on the blockage 2 m away from the emitter.

The permendur strips were attached to a 3 mm thick pipe with an inner diameter of Ø37 mm. The distances between the sensors and the blockage in the setup are as represented in Figure 3. The wave must travel 3 m (from the start of the transmission) before it reaches the receiver after bouncing on the blockage. This translates into a delay of ≈950 µs from the instant the emitter is electrically excited (Figure 4).

Figure 4 clearly shows the signal obtained due to the echo generated in the blockage, but other propagation modes L(0,2) with higher speed (5200 m/s) may appear that are not sufficiently attenuated due to the small distance to the blockage.

## 3. Magnetostrictive Sensor for Partial Blockage Detection

Section 1 discussed how to detect the position of a blockage in the pipe using torsional waves. Additional information can be found in [27].

Using the same operating principle, a magnetostrictive sensor was proposed to not only identify the position of the blockage, but also to distinguish whether the blockage covered the whole pipe section (total blockage) or only part of it (partial blockage). Although precise quantification of the density of the blockage was not pursued at this time, the variation in the attenuation of the torsional wave used to detect the plug also allowed us to distinguish materials with different densities (such as silicone and cement).

Identifying whether the blockage is total or partial is especially interesting in order to be able to remove the salt blockage without having to cut the pipe, because knowing its exact position will condition the heating process. It is important to point out that when there is total blockage, the heating cannot be carried out from its center as the change in the density of the salt during the melting process is abrupt and an overpressure appears that can even break the pipe. This situation might lead to the expulsion of low-viscosity, high-heating molten salt, endangering the working staff in the surrounding area. Having an estimate of whether the blockage is total or partial significantly reduces the risk of breaking the pipe, and allows the melting process to be accelerated in the case of partial blockages.

**Figure 5:**
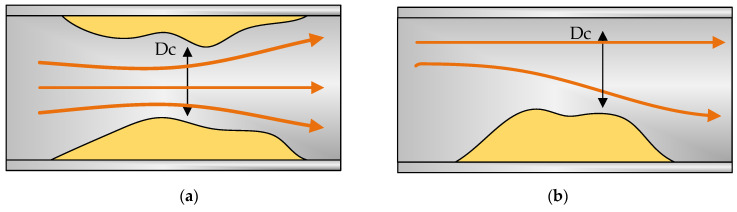
Partial blockage: (**a**) the salt has solidified all around the entire surface of the pipe, (**b**) the salt has been deposited on a specific area of the pipe. Both blockages define the same section.

Therefore, regarding blockage detection, two different possibilities can be assumed: total and partial blockage. In the second case, two main subcategories can be distinguished, as shown in Figure 5.

This work was oriented to determine the position of the blockage and whether it was total or partial using the same signal. To do so, the emitter generates an ultrasonic wave to detect the echo (and hence the position) and also identify the type of blockage.

**Figure 6:**
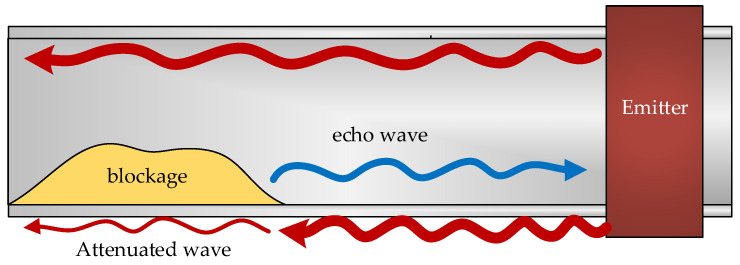
Blockage detection. Once the ultrasonic wave reaches the blockage, it produces an echo that can be used to determine its position, but the wave continues, with certain attenuation, in the same direction. The attenuation introduced by the blockage depends on the material.

The type of partial blockage in Figure 5a is easier to detect even if longitudinal waves are used, since it is in contact with the entire periphery of the pipe. We will find greater difficulty, however, with the partial blockage of Figure 5b. In this case, a longitudinal wave might not detect the area where the plug is located unless the EMAT is capable of emitting along the entire surface of the pipe (Figure 6).

The generated waveform is attenuated when it passes through the section where there is a blockage (Figure 6). This attenuation depends on the material, but also on the position of the blockage and/or the part of the pipe section affected by the blockage. These are all important points in the analysis as they allow for the position of the blockage to be detected.

**Figure 7:**
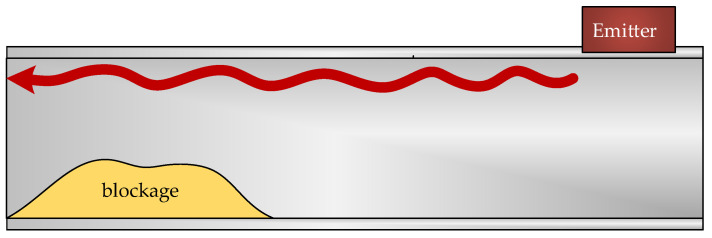
Blockage detection. The guide wave does not collide with the blockage due to its location.

The different possible configurations of the clog have important consequences in the type of ultrasonic wave that should be selected. Longitudinal (or compressional) and shear (or transverse) waves can propagate through solid materials. However, assuming the presence of a partial blockage, the use of directional waves may cause problems in its location (Figure 7).

**Figure 8:**
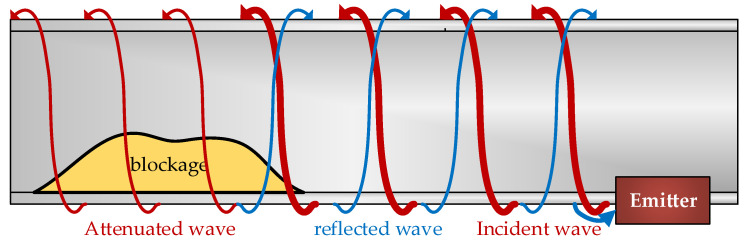
Blockage detection. The torsional wave travels along the pipe until it finds the blockage, which attenuates the propagation and gives rise to a reflected wave.

Torsional waves offer some benefits compared to compressional or transverse waves because the displacement of particles is in the circumferential direction (Figure 8). Furthermore, the propagation characteristics of torsional mode T(0,1) presents a constant velocity without any other axially symmetrical torsional mode in the used bandwidth (hundreds of kHz). Additionally, since transverse waves cannot propagate through liquids or gases, they are not affected by the liquids inside the pipe. Therefore, torsional mode T(0,1) is not dependent on the frequency used and is a non-dispersive mode at all frequencies, presenting only tangential displacement along the thickness of the pipe wall. These advantages make torsional waves T(0,1) particularly interesting for detecting partial plugs in pipes.

Therefore, torsional waves can generate an ultrasonic vibration that travels along the surface of the pipe with tangential displacement. Due to the constant velocity characteristic of this torsional wave, it is easy to calculate the position of the salt blockage by measuring the time from emission to reception of the echo. However, this is not all. The wave also provides information of the size and density of this blockage, thanks to the attenuation suffered in the section where the blockage is located.

**Figure 9:**
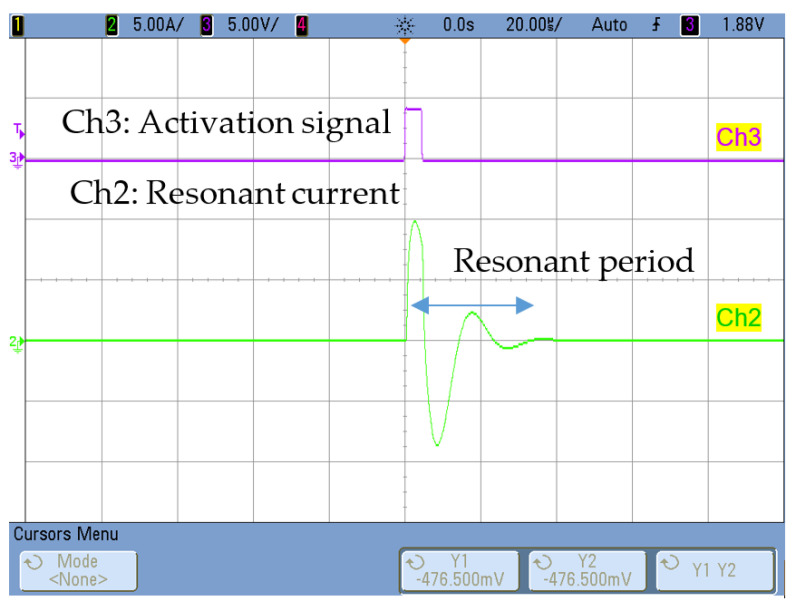
Resonant current at the converter (Iac). Ch3 [5V/div], Ch2 [5A/div].

The transmitter activation circuit remained the same as described in Section 2: a full bridge that applied a 5-µs single voltage pulse of maximum 200 V to the transmitter winding (N = 50), giving rise to a resonant current whose amplitude can be controlled with the converter voltage (Figure 9). In this case, the period of the resonant pulse has been reduced to avoid the overlap between the activation and the pulse reception.

**Figure 10:**
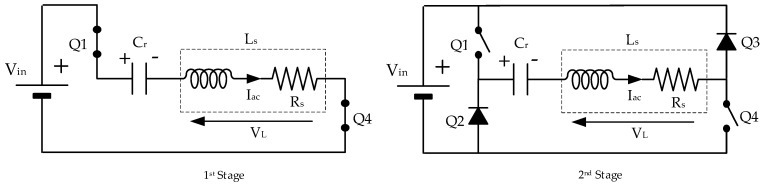
First two stages during the pulse generation.

Generating the power pulse in the full bridge inverter is simple. Transistors Q1 and Q4 of one of the diagonals are simultaneously activated during 5 µs to produce the voltage pulse across the resonant stage (Figure 10, 1st Stage). One half-period later, both transistors are switched off and the resonant current flows through the body diodes of transistors Q2 and Q3, thus charging the resonant capacitor, Cr, with a positive voltage (Figure 10, 2nd Stage). This second stage finishes when the resonant current becomes zero and tries to invert its direction. Then, the body diodes of transistors Q1 and Q4 allow the resonant current, Iac, to flow in the opposite direction, thus discharging Cr. This process goes on until both the resonant capacitor and the inductor are completely discharged (Figure 10). 

The selection of the resonant capacitor is mainly oriented to obtain a sinusoidal resonant current. For the experimental setup, the values of the resonant elements were Cr = 160 nF, Ls = 54 µF, and Rs = 7.5 Ω.

### 3.1. Torsional Wave Attenuation 

**Figure 11:**
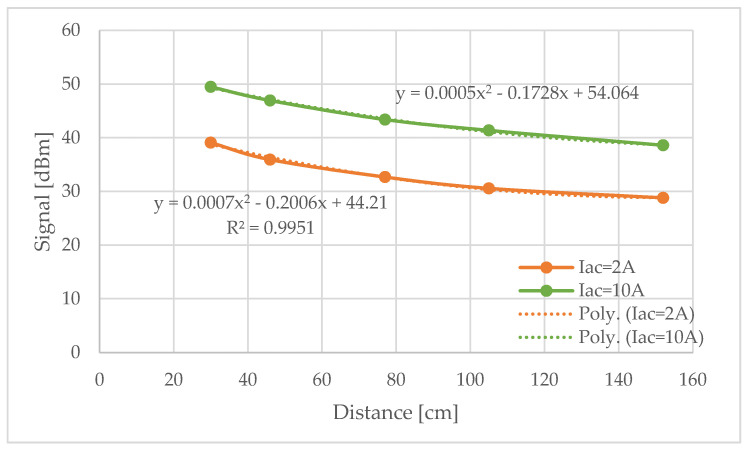
Experimental amplitude of the torsional wave signal as a function of distance (in dBm).

Driven by the above-mentioned converter, the EMAT implemented generates torsional waves. These waves suffer attenuation (A) as they travel along the aluminum pipe with a Ø37 mm inner diameter. Therefore, the amplitude of the signals detected by the receiver decreases with distance (d) to the emitter. Focusing on the maximum voltage received by the sensor, we can represent its value in dBm as a function of distance (Figure 11). Two different values for the excitation current in the transmitter (Iac) were studied. Figure 11 shows the experimental results obtained in both cases. A mathematical expression corresponding to the results obtained can also be obtained. Equation (1) allows us to determine the amplitude of the signal that reaches the receiver as a function of the distance to the emitter for the case Iac = 10 A.

(1) $$S\left[dBm\right]=20\cdot log\left(\frac{Vo}{1\;mV}\right)=0.0005\cdot d^{2}-0.1728\cdot d+54.064$$

**Figure 12:**
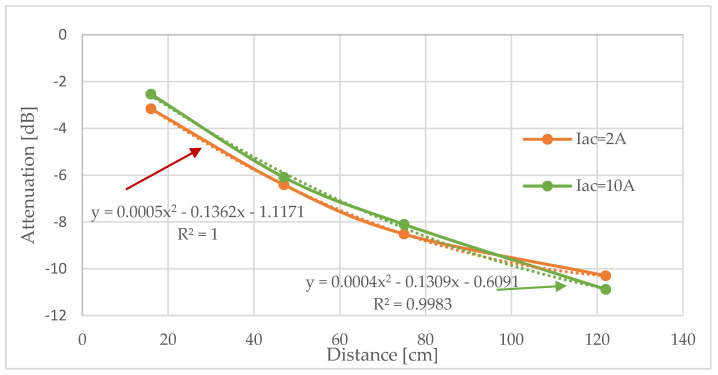
Experimental torsional wave attenuation as a function of the distance.

Once the amplitude of the signals received is known (*S*), the attenuation (*A*) of the aluminum pipe can be determined in dB. This attenuation was measured at different distances (*d*) and for different Iac currents. As expected, the attenuation did not depend on the input current, Iac, which defines the input signal (Figure 12).

**Figure 13:**
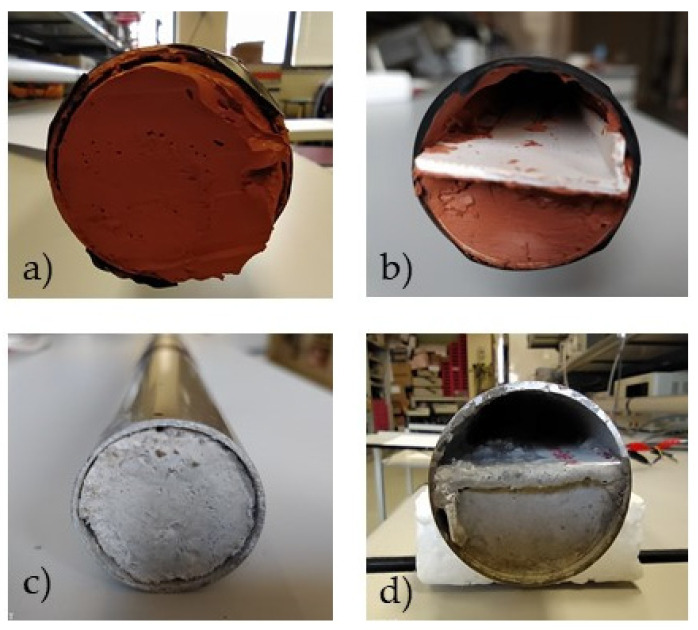
Blockages used: (**a**) and (**b**) total and partial blockages of silicon, (**c**) and (**d**) total and partial blockages of cement.

The correlation expressions obtained had almost the same coefficients regardless of the current used to generate the current pulse. The polynomial expression that best approximates the experimental results is:

(2) $$A\left[dB\right]=20\cdot log\left(\frac{Vo}{Vi}\right)=0.0005\cdot d^{2}-0.1362\cdot d-1.117$$

where *Vo* and *Vi* represent the signal at the receiver and the signal at the transmitter, respectively. Since the materials deposited on the inner surface of the pipe introduce additional attenuation, an estimation of the blockage nature can be obtained. This assumption was further analyzed by considering four different blockages. Two were made with cement and the other two with silicone. In both cases, a partial blockage and a total one were created (Figure 13).

As already mentioned, the use of molten salt compromises safety due to the high temperatures required for its fusion. It must be noted that molten salt is a material with a high calorific value and any splash could pierce the human body. To facilitate the tests carried out, cement was used instead, as the densities of cement and molten salt are similar. On the other hand, to check whether the technique tested was suitable to distinguish materials with different densities, another material with a lower density was also used, which was silicone in this case. By doing so, it is possible to compare the results when using materials of different densities. As explained later, it is relatively simple to differentiate cement blockages from silicone blockages, with signals up to four times larger (expressed in dBm) being obtained with the silicone partial plug compared to the ones obtained with the cement partial plug.

Initially, the attenuation caused by the plugs shown in Figure 13 was analyzed by mere comparison between the maximum amplitude measured in the receiver and the signal generated in the transmitter by a 10-A_pk_ current (Figure 9). In order to reduce the noise in the received signal (*y_r_*), a synchronous averaging process (*S_m_*) was used as a filter Equation (3).

(3) $$S_{m}\left(t\right)=\frac{1}{N}\overset{N-1}{\underset{n=0}{\sum }}y_{r}\left(t+nT\right)$$

where *N* is the number of average and *T* is the signal period.

**Figure 14:**
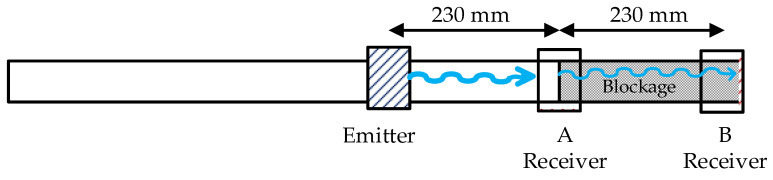
Workbench used for the blockage characterization.

The transmitter and the receiver had the same structure: four permendur strips at 45° surrounded by a 50-turn solenoid. Two permanent magnets provided DC magnetic polarization for the permendur strips (Figure 1). The workbench used was similar for all type of blockages. However, while the position of the emitter was always the same, the receiver was placed at two different distances from the emitter: 23 cm and 46 cm (positions A and B). The first position corresponded to the beginning of the blockage, whereas the second one defined the end of the pipe and the blockage (Figure 14).

According to Equation (1), the attenuation introduced by the 230 mm long pipe itself resulted in a signal of 50.3 dBm. The measured signal had a very similar value, although was slightly lower due to the incipient presence of the obstruction (see Table 2, position A below).

Regarding the attenuation introduced by the materials used, a difference of 10 dBm was detected in position A.

In position B, and given that cement has the highest density of the two materials considered, it generated a much greater attenuation than that shown by silicone (Table 2). In this position, the received signal could reach a difference of 30 dBm if the blockage is partial. The total cement plug of 230 mm attenuated the acoustic waveform so much that the sensor in position B could not detect it.

Accordingly, it can be seen that in any of the positions occupied by the receiver, the difference in the densities of the materials used in the blockages is easily detectable.

Due to the difficulty of accurately controlling the position of the receiver, a tolerance of 1 dBm was detected in the signal received at position A when comparing the partial and total blockage of the same material.

On the other hand, in position A, the signal received does not depend so much on whether the blockage is partial or total, since the receiver also covers an area without a plug.

**Figure 15:**
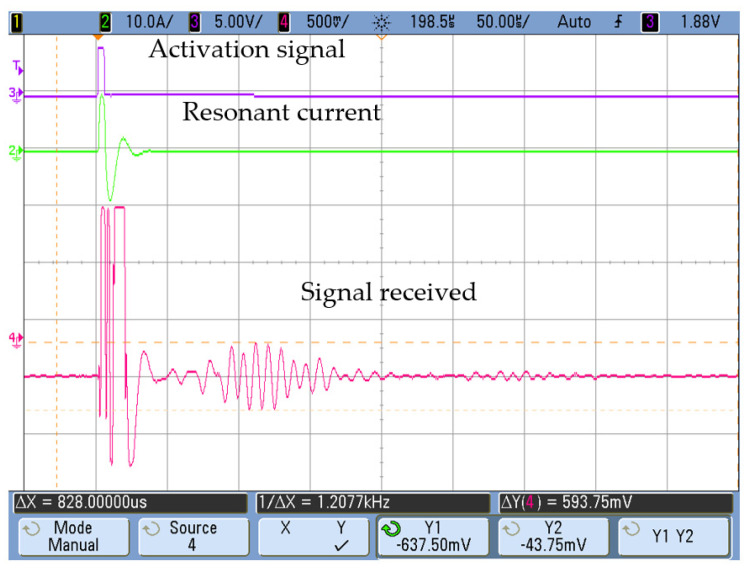
Signal received with a total silicone blockage (Figure 13a) at position A.

**Figure 16:**
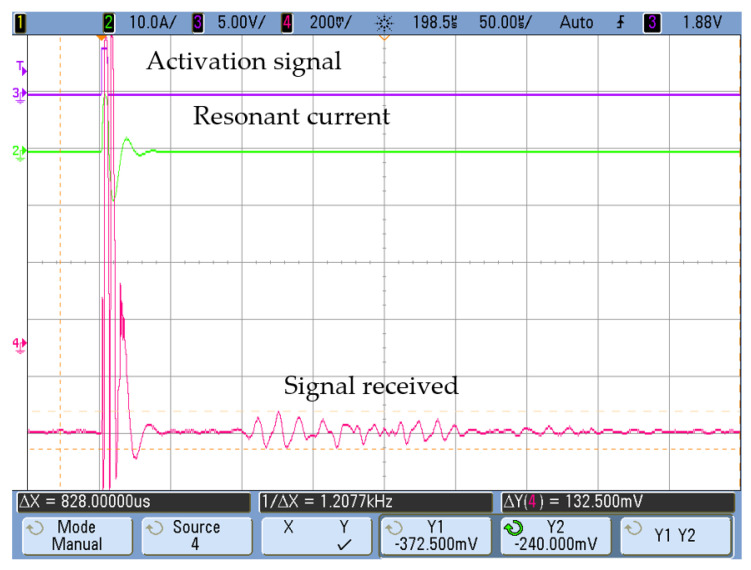
Signal received with a total silicone blockage (Figure 13a) at position B.

As an example of the measurements obtained, Figure 15 and Figure 16 show the signal received at positions A and B with a silicone blockage. In both cases, the activation current in the transmitter was the same: a 10-A_pk_ resonant current obtained with a single voltage pulse. It can be seen that there was a significant amplitude reduction in the received signal due to attenuation of the plug, depending on the receiver position (A or B).

The EMAT shown in Figure 1 (which was used to obtain the results in Table 2) provided similar values for both partial and total blockages when their length was of the order of hundreds of mm. The scheme in Figure 14 helps us to distinguish materials with different densities such as silicone and cement. Distinguishing the type of blockage (total or partial) was more difficult.

### 3.2. Torsional Wave Correlation 

In certain cases, such as the one with a cement blockage and the receiver in position B, the signal amplitude at the receiver is difficult to measure. Even after synchronous averaging, the signal-to-noise ratio must be further improved. For this purpose, two digital filters were applied to the signal obtained in the receiver, *vr*. The first was a high-pass filter with a cut-off frequency of 1 kHz to remove the DC component. The second was a matching filter, which correlated the signal with a sinusoidal mask, *vp*, of 10 µs period (Equation (4)). This mask is normalized, so the sum of all of its points equals the unit. The number of points of the mask, np, depends on the signal sample rate.

(4) $$vp\left[n\right]=abs\left[\frac{sin\left(2\pi \frac{n}{np}\right)}{\sum_{k=1}^{np}sin\left(2\pi \frac{k}{np}\right)}\right]$$

where *np* is the number of points per period; since the sampling frequency of the experiment was 2 MHz, *np* was fixed to 20. Accordingly, *n* varies from 1 to np.

The *C*[*m*] correlation was obtained after moving the mask along the measured signal (Equation (5)). It was then compared with a voltage of 1 mV (Equation (6)), which allows us to obtain its value in dBm by simply taking logarithms in (Equation (6)).

(5) $$C\left[m\right]=\overset{np}{\underset{n=1}{\sum }}\left\{vp\left[n\right]\cdot abs\left(vr\left[n+m\right]\right)\right\}$$

(6) $$vr_{m}^{*}=\frac{C\left[m\right]}{1mV}$$

**Figure 17:**
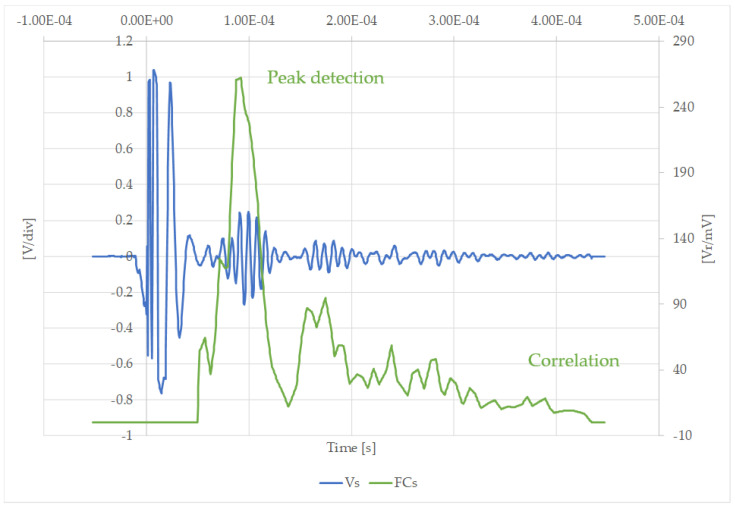
Measured, Vs [V/div], and filtered signal, FCs [Vr/mV], after using the correlation filter in the case of a partial silicone blockage at position A.

Now, using Equation (6), the amplitude of the received signal is more clearly identified. Figure 17 shows the measurements obtained in the case of a partial blockage of silicone with the receiver at position A, with and without the matching filter.

**Figure 18:**
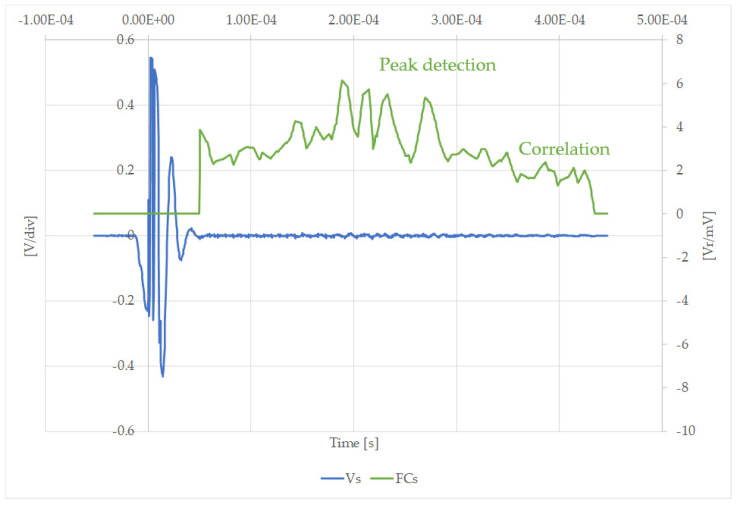
Measured, Vs [V/div], and filtered signal, FCs [Vr/mV], after using the correlation filter in the case of a total cement blockage at position B.

Figure 17 shows how evident the amplification obtained by the filtering process is in the case of an easily measured signal. Figure 18 illustrates that the filtering process is essential when the maximum amplitude of the received signals is more difficult to obtain. It was therefore confirmed that a mathematical post-processor is needed to improve the signal-to-noise ratio. For this purpose, a matching filter was implemented.

All the signals measured and shown in Table 2 were processed with the described correlation filter, giving rise to the results summarized in Table 3.

The signal received in position B when the plug was made of cement had a very low amplitude, so the accuracy of the measurement was also low.

**Figure 19:**
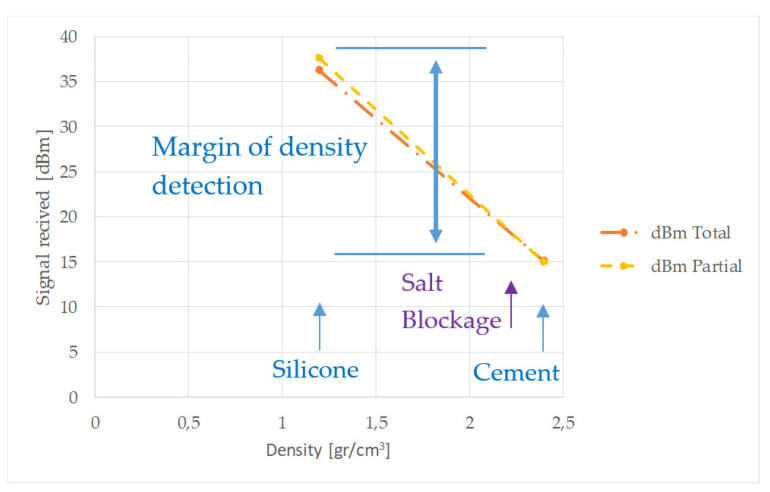
Transducer response versus material density with total and partial blockage (position B).

Identification of blockage density was also analyzed. The results shown in Figure 19 suggest a wide detection range of blockages with different material densities. It is true that by using only two types of materials (cement and silicone), it is not possible to know the exact evolution of the signal with respect to the density. However, other experiments with cylindrical bars surrounded by soil [31] propose an almost linear relationship between density and attenuation for torsional waves T(0,1); that is why a straight line was plotted in Figure 19, joining the two results obtained in our tests. Accepting this linearity, the ability of the sensor to discern densities between the typical value of the plugs caused by molten salt (similar to that of cement) and other less dense materials can be assumed.

### 3.3. Portable Magnetostrictive Sensor

In solar thermal power plants, the pipelines are surrounded by thermal insulation, which makes it difficult to use transducers such as those shown in Figure 1. To solve this problem, a modification of the sensor was proposed [20] that reduced the number of permendur strips used to one.

**Figure 20:**
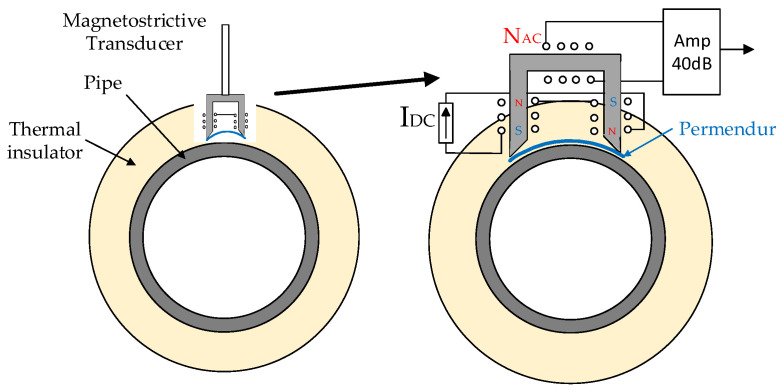
New magnetostrictive transducer.

With this technique, the signals obtained in the receiver had a lower amplitude, thus requiring the use of amplification and filtering stages like the ones described above. The geometry of the sensor (Figure 20) allows the thermal insulation of the pipe to be pierced and the metal to be accessed as if the sensor were a thermal probe.

The use of a portable sensor as shown offers additional advantages in the case of partial blockages. Since it is not necessary to embrace the entire pipe, this sensor can be positioned on the pipe surface where the plug is located or where it is free of a plug. In this way, it is possible to detect which area of the pipe surface has a partial plug and where it is located.

In this portable sensor, the measurement of the vibration in the strip will be carried out with a U-shaped magnetic core that adapts to the dimensions of the strip. The magnetic core incorporates two windings, one for continuous magnetic polarization (I_DC_ = 5 A), and one for the measurement of the alternating signal caused by the vibration of the permendur strip (N_AC_ = 50).

The measurement process will start by magnetizing the permendur strip with a direct current of 5 A to achieve the necessary polarization. This current is then removed and the vibration received through the AC winding is measured through an amplifier (Figure 20). It should be noted that, as indicated above, when there is a partial blockage of the pipe, the position of the permendur strip with respect to the plug affects the result.

**Figure 21:**
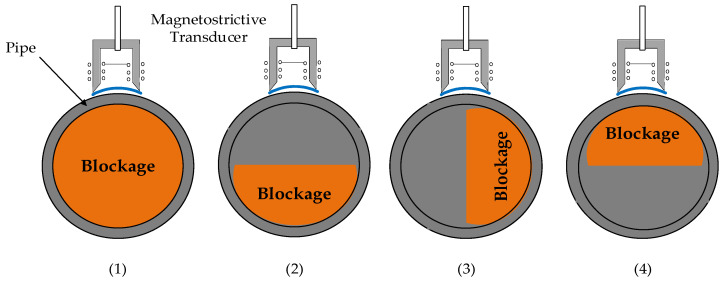
Positioning of the magnetostrictive transducer with respect to the clog.

Figure 21 shows four possible situations of the permendur strip and the sensor with respect to a partial blocking. These four alternatives, and positions A and B from Figure 14, are considered in Table 4, which summarizes the experimental results obtained with a silicone and cement clog.

The ability to identify partial plugs largely depends on the density of the plug. In the case of silicone (1.2 g/cm^3^), the measurement obtained with a blockage in position 4 compared to the measurement in the section where there is no blockage (position 2) showed variations of 22%, which is a significant margin. If we compare the signal received when there is half a section of the silicone plug and the one obtained when there is no plug, we can see variations of up to five times. This allows for a wide margin for detecting plugs with sections smaller than 50%.

This situation is much more pronounced if the density of the plug is greater, as in the case of cement (2.4 gr/cm^3^) or molten salt, which also has a high density value (2.2 gr/cm^3^).

**Figure 22:**
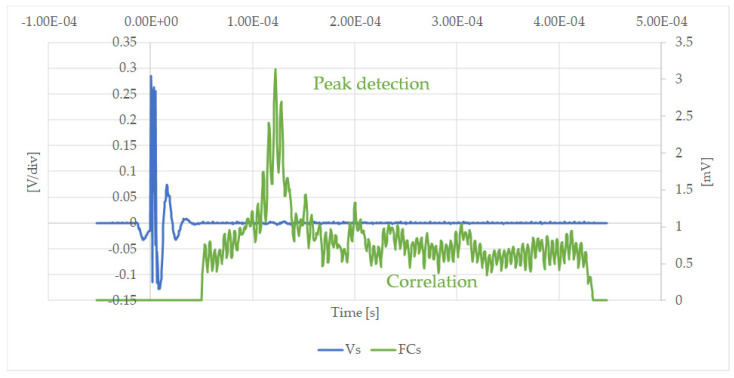
New sensor results: Experimental signal in the receiver, Vs [V], and filtered signal, FCs [mV] after using the correlation filter with a partial cement blockage in position B-2.

**Figure 23:**
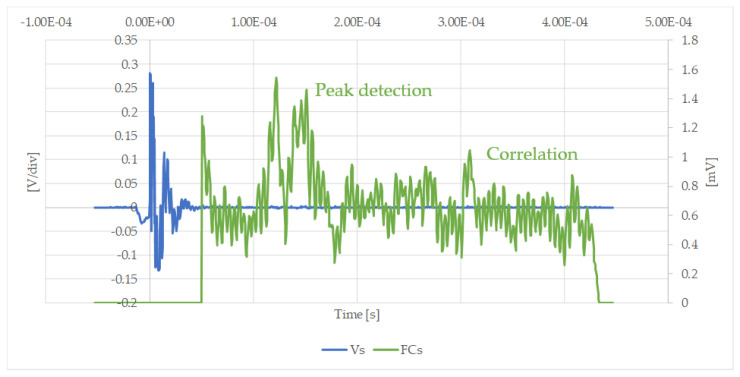
New sensor results: Experimental signal in the receiver, Vs [V], and filtered signal, FCs [mV] after using the correlation filter with a partial cement blockage in position B-3.

**Figure 24:**
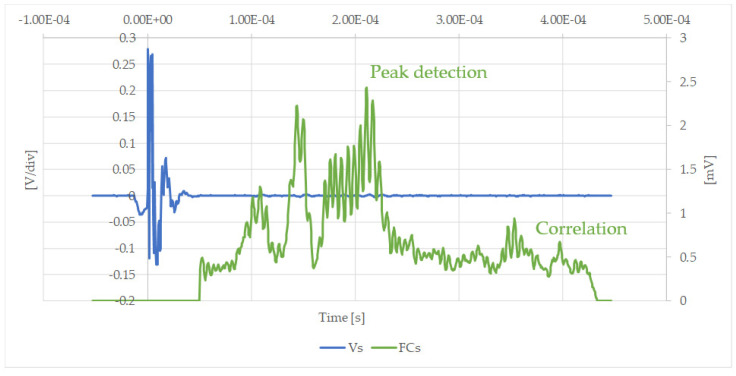
New sensor results: Experimental signal in the receiver, Vs [V], and filtered signal, FCs [mV] after using the correlation filter with a partial silicone blockage in position B-4.

**Figure 25:**
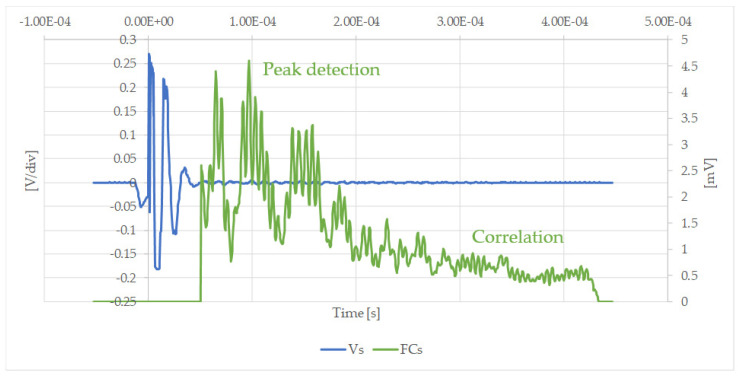
New sensor results: Experimental signal in the receiver, Vs [V], and filtered signal, FCs [mV] after using the correlation filter with a partial silicone blockage in position A-4.

The curves in Figure 22, Figure 23, Figure 24 and Figure 25 are some examples of the experimental results presented in Table 4. They show the comparison of the individual signals obtained from the sensor (in volts) and the final response after being processed according to Equation (5). Since the attenuation introduced by the pipe itself is known, any additional attenuation of the signal corresponds to the presence of a plug and, depending on the amplitude of this attenuation, it is possible to identify the characteristics of the blockage.

Again, it is important to emphasize the need to introduce a mathematical filter to improve the signal-to-noise ratio. In this paper a matching filter was used, but other alternatives have also been used in the literature [20,21,22].

In the case of a partial silicone plug, due to its low density (1.2 g/cm^3^), the amplitude obtained in position A, the point where the plug begins, makes it difficult to identify the area of the pipe in which it is located (position A-2 or A-4). The comparison may be more evident when comparing the plugs between position A and B.

## 4. Discussion

The detection of clogs and defects in pipes is a problem that has been widely studied, leading in many cases to EMAT-based solutions. The design of the sensors depends on each particular situation but, in most cases, these transducers are placed around the pipe. However, in the specific case of solar thermal power plants, where molten salts are used as the heat transfer fluid, the presence of clogs is a problem that requires a minimally intrusive solution. In this type of system, two questions arise: on one hand, the detection of the position of the blockage in the pipe, and on the other hand, a closer study of its borders, geometry, and consistency to evaluate the best method to re-melt the salts. Being able to distinguish whether a blockage is total or partial and accurately identify the affected area is very important when defining how to heat the pipe. The goal is always to avoid pipe bursting due to an undesirable pressure increase in the re-liquefied salt, which would mean a high physical hazard for the operators.

The presence of a thick layer of thermal insulating material around the pipe complicates access to the pipe. Removing that layer to apply the electromagnetic acoustic sensor is a serious drawback. For this reason, an EMAT sensor has been developed that avoids the need to remove all the insulation around the pipe. This sensor is small enough to be inserted through the thermal insulator with minimal intrusion, just as one would with a thermal probe. In this way, it is not necessary to uncover an entire section of the pipeline, but simply drill a hole in it to insert the sensor. Despite its small size, this sensor provides the double functionality desired: the detection of the clog position, and once it has been located, the identification of its limits and whether it is total or partial. Sensor performance was supported by experimental results obtained in the lab.

## 5. Conclusions

The use of magnetostrictive sensors has been an important technique in the condition monitoring of pipelines. Several sensors have been developed and adapted to different problems such as the detection of defects on the internal face of the pipes. In this paper, a magnetostrictive sensor was developed and experimentally verified for the location of clogs and the estimation of whether the blockage was total or partial in pipelines through which molten salts circulate.

A relevant aspect of the proposed sensor is the possibility of identifying the clog position in a minimally intrusive way, since it does not require the total removal of the thermal insulation layer that surrounds the pipeline, but only the boring of holes at specific points.

This aspect is especially relevant in solar thermal power plants, in order to identify the surface affected by the plug and proceed to melting it safely, avoiding possible breaks due to overpressure. Nowadays, the alternative used involves obtaining an image using radiometric techniques [26], which require strict security protocols and qualified personnel.

The experimental results obtained with two materials (cement and silicone) provide information on the attenuations introduced in each case according to the size, position, and type of material of the clog. With this information, conclusions can be drawn related to the density of the clog, whether it is total or partial, and the position of the partial clog in the pipe section.

It should be noted that the developed transducer showed a lower amplitude in the received signals, which makes mathematical post-processing of the information obtained necessary to improve the signal-to-noise ratio. The access to a limited section of the pipe affects the magnitude of the reception, which obliges a suitable post-processing algorithm to improve the signal-to-noise ratio.

In light of the experimental results obtained, a possible alternative to the use of radiometric methods is offered. This is very interesting because, although these radiometric methods are not intrusive, they have a high cost and exceptional safety conditions.

## Figures and Tables

**Table 1:** Elongation coefficient of the analyzed magnetostrictive materials.

A. Elongation Coefficients	
Material	∆L/L
Nickel	−33 × 10^−6^
45 Permalloy	27 × 10^−6^
Vanadium Permendur	70 × 10^−6^
Steel	21 × 10^−6^

**Table 2:** Signal measured in dBm depending on the type of blockage and the position of the receiver.

Blockage	Position A	Position B
Silicon (total)	49.42 dBm	36.4 dBm
Silicon (partial)	48.27 dBm	38.58 dBm
Cement (total)	38 dBm	No signal measured
Cement (partial)	38.27 dBm	10 dBm

**Table 3:** Amplitude of the filtered signal (in dBm) for different types of blockage and different positions of the receiver.

Blockage	Position A	Position B
Silicon (total)	49.1 dBm	36.16 dBm
Silicon (partial)	48.36 dBm	37.57 dBm
Cement (total)	38.1 dBm	15 dBm
Cement (partial)	38.54 dBm	14.9 dBm

**Table 4:** Amplitude of the filtered signal (in dBm) for different types of blockage and different positions of the receiver.

Blockage	Position A			Position B		
Silicone (total)	17.2 dBm			10.29 dBm		
	**Position** **(2)**	**Position** **(3)**	**Position** **(4)**	**Position** **(2)**	**Position** **(3)**	**Position** **(4)**
Silicone (partial)	14.25 dBm	13.4 dBm	13.0 dBm	8.6 dBm	7.3 dBm	6.7 dBm
Cement (partial) ^1^	13.9 dBm	10.9 dBm	7.5 dBm	9.8 dBm	3.78 dBm	0.42 dBm

^1^ The distance between position A and B was reduced to 100 mm for the cement blockage.
